# Isolated left pulmonary artery hypoplasia

**DOI:** 10.11604/pamj.2020.36.14.22539

**Published:** 2020-05-12

**Authors:** Imen Touil, Nadia Keskes Boudawara, Soumaya Bouchareb, Jalel Knani, Leila Boussoffara

**Affiliations:** 1 Pulmonology Department, Tahar Sfar Hospital, Mahdia, Tunisia

**Keywords:** Hypoplasia, artery, lung, adult

## Abstract

Pulmonary artery hypoplasia is a rare malformation of childhood that is usually associated with cardiac abnormalities. In the absence of these cardiac malformations it is discovered later when respiratory signs appear. It was a 56-year-old patient who had been referred for dyspnea with cough. The physical examination was normal. Chest X-ray, thoracic computed tomography (CT) scan and echocardiography suggested the diagnosis of hypoplasia of the left pulmonary artery without associated cardiac malformations. The early diagnosis of hypoplasia of the pulmonary artery allows the close follow-up of these patient and the planning of an adequate management.

## Introduction

Unilateral pulmonary artery agenesis with lung hypoplasia is one of the rare congenital developmental anomalies of the lung. In fact, its prevalence was estimated at 1/200,000 [1]. There are no specific symptoms for this malformation. While some of the congenital lung lesions are diagnosed in neonates or in later periods of life, others are asymptomatic and may be diagnosed during the childhood or adult periods accidentally [2]. Prognosis depends on the extent of hypoplasia, what the underlying cause of the condition is and the presence of other congenital abnormalities. This condition is therefore managed in a number of different ways.

## Patient and observation

**Figure 1:**
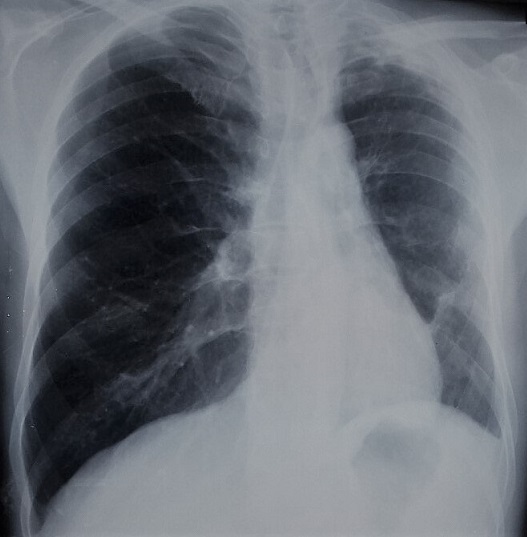
Front chest x-ray: retraction of the left pulmonary field, reduction of the left hilum and hyperinflation of the right side

**Figure 2:**
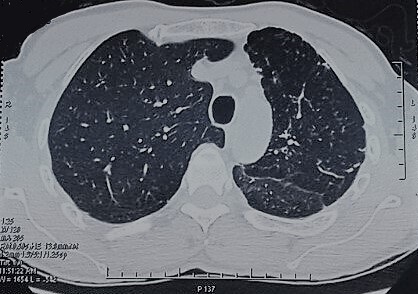
Chest CT parenchymal section: hernia of the right lung, reduction of the left lung volume with air trapping of the left upper lobe without intra-parenchymal or bronchial abnormality

**Figure 3:**
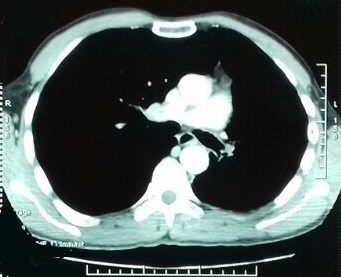
Mediastinal section thoracic CT scan: reduction in the diameter of the left pulmonary artery with poorly developed collateral bronchi

A 56-year-old male patient presented with complaints of progressive dyspnea on exertion associated with dry cough for two months. It was a former smoker with no comorbidities. He confirmed progressively worsening dry and hacking cough. The dyspnea was grade I modified Medical Research Council (mMRC). However, there was no history of fever, wheeze, chest pain, anorexia, or weight loss. On clinical examination, he maintained saturation of 98% of oxygen with no crackles or wheezing. The other clinical examinations and laboratory tests were normal. On evaluation, chest X-ray (Figure 1) revealed retraction of the left pulmonary field and hyperinflation of the right side. A contrast enhanced computed tomography (CT) of the chest was performed. It confirmed the hypoplasia of the left pulmonary artery. In fact, it was reduced in diameter and length compared to that on the right with poorly developed collateral branches including those of the lower lobe. On chest CT, there was an air trapping of the superior lobe of the left lung (Figure 2, Figure 3). No signs of malformations were noted on transthoracic echocardiography. However, there was pulmonary hypertension (pulmonary artery pressure (PAP) = 55 mm Hg), dilated right ventricle and minimal tricuspid valve regurgitation. Pulmonary functions tests were compatible with obstructive lung disease: FEV11,4L (49% pred) and FEV1/FCV = 52% pred. The patient, with the above findings, has been under follow-up with a diagnosis of left pulmonary artery and a chronic obstructive pulmonary disease (COPD). Without considering a surgical intervention, patient used a long-acting bronchodilator and inhaled corticosteroid therapy with clinical and radiological monitoring.

## Discussion

Unilateral hypoplasia of a pulmonary artery (HPA) is caused by a malformation of the sixth aortic arch of the affected side during embryogenesis [1]. This congenital malformation is classified into two types which is as follows: (1) agenesis: proximal interruption of pulmonary arteries (PA); (2) hypoplasia: pulmonary arteries remained rudimentary. It has also been perceived that most of the cases affect the left PA [2]. This condition has been reported with a variety of cardiac malformations and usually presents with symptomatic disease. The associated cardiac malformations include tetralogy of Fallot [3], atrial septal defect, coarctation of aorta, right aortic arch, and Eisenmenger's syndrome [4]. In the great majority of the cases in the literature, patients were diagnosed in infancy or childhood. In fact, only a limited number of patients appear to remain asymptomatic until adulthood [5]. In the case reported here, the patient had a left-sided pulmonary artery hypoplasia without the associated congenital cardiac anomalies which could explain the maintenance of an asymptomatic state.

According to previously published reports, presenting symptoms in adult patients can be variable such as exertional dyspnea or limited exercise intolerance (40%), hemoptysis (20%), recurrent pulmonary infections (37%) and pulmonary hypertension (25%) [6, 7]. It has been proposed that hemoptysis could be caused by the collaterals arising from bronchial, subclavian, subdiaphragmatic and intercostal arteries which supplied the affected lung. On the other side, alveolar hypocapnia can cause bronchoconstriction, while impaired mucociliary clearance and diminished delivery of inflammatory cells may contribute to the high incidence of respiratory infections [8]. Chest X-ray of agenesis hypoplasia may give clue of the pulmonary agenesis. It reveals a reduction in the volume of hemithorax, an elevation in the diaphragm, reduced intercostal spaces and mediastinal shift in the affected side. Compensatory hyperinflation may be seen on the opposite side. Contrast enhanced computed tomography (CT) is almost definitive for the diagnosis: it shows the rudimentary affected PA. This noninvasive test provides detailed morphological information and determines the presence of cardiovascular malformations [8]. Usually, CT angiography confirmed the findings of CT. This test is suitable for evaluation of the collateral circulation, and also contributes greatly to treatment. In fact, in cases of repeated hemoptysis, this test is used to evaluate the collateral circulation, and the blood vessels responsible for this hemoptysis are embolized [9]. Pulmonary angiography remains the gold standard for diagnosis of vascular pulmonary malformations [10]. This invasive test imaging is not usually performed in order to make a definitive diagnosis of hypoplasia PA. Perfusion scintigraphy can be also performed, it shows typically normal ventilation but no perfusion on the affected side. Other evaluation techniques, including cardiac catheterization and magnetic resonance imaging, have also been used to diagnose this condition. However, as this case illustrated, a CT scan is quite useful in revealing the features of this disorder.

In addition, echocardiography should be performed in order to exclude cardiac malformations associated to this condition. In our case, this test reveals a pulmonary hypertension. Many patients with hypoplasia PA can remain asymptomatic for a long time, but the development of pulmonary hypertension may preclude long-term survival. In fact, the mortality rate of this condition remains 7%-8% [11]. Unfortunately, there are no guidelines or consensus regarding treatment. Surgical management can only be attempted in accompanying cardiac and vascular anomalies, in recurrent hemoptisis attacks, or in persistent lung infections and bronchiectasis [8]. Patients require close follow-up of pulmonary hemodynamics as well as continued medical management for pulmonary hypertension such as: calcium channel blockers, endothelin receptor antagonists and intravenous prostacyclin. Selective embolization of the collaterals may be done to control massive hemoptysis.

## Conclusion

Left pulmonary artery hypoplasia is a rare congenital malformation. Computed tomography is generally sufficient for definitive diagnosis. Prognosis depends on many complications including mainly severe pulmonary hypertension and pulmonary hemorrhage.

## Competing interests

The authors declare no competing interests.
